# Risk indicators for severe impaired oral health among indigenous Australian young adults

**DOI:** 10.1186/1472-6831-10-1

**Published:** 2010-01-27

**Authors:** Lisa M Jamieson, Kaye F Roberts-Thomson, Susan M Sayers

**Affiliations:** 1 Australian Research Centre for Population Oral Health, The University of Adelaide, Adelaide, South Australia 5005, Australia; 2 Menzies School of Health Research, Charles Darwin University, Darwin, Australia

## Abstract

**Background:**

Oral health impairment comprises three conceptual domains; pain, appearance and function. This study sought to: (1) estimate the prevalence of severe oral health impairment as assessed by a summary oral health impairment measure, including aspects of dental pain, dissatisfaction with dental appearance and difficulty eating, among a birth cohort of Indigenous Australian young adults (n = 442, age range 16-20 years); (2) compare prevalence according to demographic, socio-economic, behavioural, dental service utilisation and oral health outcome risk indicators; and (3) ascertain the independent contribution of those risk indicators to severe oral health impairment in this population.

**Methods:**

Data were from the Aboriginal Birth Cohort (ABC) study, a prospective longitudinal investigation of Aboriginal individuals born 1987-1990 at an Australian regional hospital. Data for this analysis pertained to Wave-3 of the study only. Severe oral health impairment was defined as reported experience of toothache, poor dental appearance and food avoidance in the last 12 months. Logistic regression models were used to evaluate effects of demographic, socio-economic, behavioural, dental service utilisation and clinical oral disease indicators on severe oral health impairment. Effects were quantified as odds ratios (OR).

**Results:**

The percent of participants with severe oral health impairment was 16.3 (95% CI 12.9-19.7). In the multivariate model, severe oral health impairment was associated with untreated dental decay (OR 4.0, 95% CI 1.6-9.6). In addition to that clinical indicator, greater odds of severe oral health impairment were associated with being female (OR 2.0, 95% CI 1.2-3.6), being aged 19-20 years (OR 2.1, 95% CI 1.2-3.6), soft drink consumption every day or a few days a week (OR 2.6, 95% 1.2-5.6) and non-ownership of a toothbrush (OR 1.9, 95% CI 1.1-3.4).

**Conclusions:**

Severe oral health impairment was prevalent among this population. The findings suggest that public health strategies that address prevention and treatment of dental disease, self-regulation of soft drink consumption and ownership of oral self-care devices are needed if severe oral health impairment among Indigenous Australian young adults is to be reduced.

## Background

In recent years, interest in the impact of oral diseases on quality of life has intensified [1]. This is due to the increased recognition that clinical oral health measures, when used in isolation, lack context without additional measures of the functional and psychosocial aspects of oral health, as well as the concerns and perceived needs of a given population [2]. This has led to the development of instruments that measure perceived impacts of oral health, which have in turn enabled greater insight into the emerging domains of oral health-related quality of life and recognised need for oral health care [3]. All instruments include assessments of three intrinsic components of oral health-associated quality of life: pain, appearance and function.

Toothache is the most common cause of pain in the mouth, often being severe enough to affect quality of life [4]. Untreated dental decay is the most common cause of toothache, although fractured teeth, exposed dentine due to wear and dental erosion may also cause pain [5]. The self-reported experience of toothache is strongly correlated with untreated dental disease [6]. Disparities in self-reported experience of toothache are well recognised, with ethnic minority groups, the financially-disadvantaged and those with less formal education being disproportionately represented [7].

The appearance of one's mouth has been reported as one of the most important features in regards to facial attractiveness [8], with associated consequences on self-image, social interaction and psychological health [9]. There are many reasons for dissatisfaction with dental appearance, including concerns about missing teeth [10], the position, alignment or spacing of teeth [11], colour of teeth or oral soft tissues [12], scarring and trauma [13], presence of oral pathology [14] or presence of prosthodontic appliances [15]. The perceived associations between dento-facial attractiveness and social traits, such as personality and social status, make dental appearance a substantial concern for many people [16].

Food avoidance is an oral health impact that may reflect functional difficulty, or which may be a consequence of discomfort or embarrassment [17]. Food avoidance is likely to reduce enjoyment of eating and affect ability to maintain a healthy nutritional status [18]. The avoidance of difficult-to-chew foods is associated with reduced body mass index and serum albumin levels [19]. Re-establishment of masticatory function in such individuals is considered an integral component of their medical health care, with the aim of improving their nutritional status and quality of life [19].

Indigenous Australians (those identifying as Aboriginal or Torres Strait Islander, or both) comprise 2.6 percent of the Australian population [20]. The Indigenous proportion of the total population increases with increasing remoteness, with 24 percent of the Indigenous population residing in remote locations compared with less than 3 percent of non-Indigenous Australians [20]. Australia's Northern Territory has the largest Indigenous population in percentage terms for a state or territory, with 31.6 percent in 2006 [20]. All other Australian states and territories have less than 4 percent of their total populations identifying as Indigenous.

There are marked disparities in the health, including oral health, of Indigenous Australians relative to their non-Indigenous counterparts. They have 15-20 years shorter life expectancy, much higher levels of cardiovascular disease, diabetes and other chronic conditions, and are more likely to experience disability and reduced quality of life due to ill health [21]. In the National Survey of Adult Oral Health, levels of untreated dental decay were more than twice as high among Indigenous Australians compared with non-Indigenous Australians [22] and in an ongoing surveillance program of services received from dental public health services, Indigenous adult public dental patients were noted as lacking appropriate preventive care [23].

The aims of this analysis are: (1) to estimate the prevalence of severe oral health impairment as assessed by a summary oral health impairment measure (including aspects of dental pain, dissatisfaction with dental appearance and difficulty eating) among a birth cohort of Indigenous Australians; (2) to compare severe oral health impairment prevalence according to demographic, socio-economic, behavioural, dental service utilisation and oral health outcome risk indicators; and (3) to ascertain the independent contribution of those risk indicators to severe oral health impairment in this population.

## Methods

### Participants and procedure

Participants were members of the Aboriginal Birth Cohort (ABC) study, a longitudinal investigation of health and behavior in a birth cohort of Australian Aboriginals. Babies were eligible for enrolment if they were live born singletons delivered at the Royal Darwin Hospital, Northern Territory, Australia between January 1987 and March 1990 to a mother recorded as Aboriginal. Of the mothers found and interviewed at baseline, 686 agreed to participate, accounting for 55 percent of potential recruits. There were no mean birth weight or gender ratio differences between those recruited and not recruited [24]. Because this was a birth cohort, sample size was not calculated.

Follow-ups were done at mean ages 5, 11 and-most recently-18 years. The Human Research Ethics Committee of the Northern Territory Department of Health and Community Services and Menzies School of Health Research (including an Aboriginal sub-committee with absolute right of veto) granted ethics approval for each assessment phase. Study members gave informed consent before participating.

In Wave-3 of the ABC study, which forms the basis of this analysis, a letter of introduction was sent to each community council explaining the purpose of the study, requesting assistance in locating a suitable space in which to work, and enquiring as to the availability of a local person to be employed as a 'locator'. A permit to visit the community was applied for and lists of potential participants were faxed through to community councils and/or health centres. Once in a given community, a number of strategies were employed to locate participants; primarily through the locally-employed locator, but also by canvassing high schools, homes, work places and recreation areas. Participants would usually be collected and dropped off by study members in a hired vehicle, and were encouraged to bring along any children or other family members. In the more urban settings, participants were canvassed in a similar manner, although there was a greater reliance on telephone calls and house calls. Snow-ball techniques were also employed. A considerable number of attempts were made to contact participants, only ceasing when the study team left the community or were given an outright refusal from a study member.

Wave-3 consisted of a range of demographic, anthropometric and social well-being items. It also included, for the first time, a dental component, which comprised a self-report dental questionnaire and dental examination.

### Summary variable for severe oral health impairment

The summary variable for severe oral health impairment variable was created by combining three items in the dental self-report questionnaire; experience of toothache, experience of discomfort due to mouth appearance and food avoidance. The questions were based on the same items implemented in the concurrently run National Survey of Adult Oral Health [22] but reworded so that items were more understandable, culturally acceptable and user-friendly. Re-worded items were discussed by members of the ABC study research team, who have extensive experience working with Aboriginal groups, and with two members of the Human Research Ethics Committee of the Northern Territory Department of Health and Community Services and Menzies School of Health Research Aboriginal sub-committee. Re-worded items were pre-tested on five Aboriginal young adults living in Darwin. Experience of toothache was assessed by asking 'Do you have any trouble with your teeth, gum or jaw right now?', with response options of 'yes' or 'no'. Experience of discomfort due to mouth appearance was assessed by asking 'Do you think your teeth are looking ok?' with response options dichotomised into 'all good' and 'some or none good'. Avoiding food because of oral health problems was assessed by asking participants 'Since the last wet, have you stopped eating some foods because they hurt your teeth?' and response options were 'yes' or 'no'. 'Since the last wet' pertains to the 'wet season' period which typically lasts from around November to March of each year in Australia's Northern Territory. For purposes of this analysis, those who answered 'yes' to the pain and function items, and 'some or none good' to the appearance item were considered to have severe impaired oral health because of oral health-related quality of life factors.

### Risk indicators

Self-report information was also sought on demographic, socio-economic, diet, dental service utilisation, dental behaviour and dental fear outcomes. Water fluoride values were not available for all communities in which participants were located, so were not included.

#### Demographic

Age, sex and location were included. Location was dichotomised into 'regional', which included participants living in the three regional centres included in the study, and 'rural/remote' which included participants living outside the regional jurisdictions.

#### Socio-economic

Source of household income was defined as 'job' (ie employment) or 'welfare' (ie unemployment or various government welfare programs). Because conventional socio-economic measures do not have the same meaning in an Australian Aboriginal context, particularly in remote communities where employment is scarce and education opportunities limited, the socio-economic position of participants was also assessed using household size and car ownership. Household size was assessed by the question 'How many people stayed in your house last night?' while car ownership was measured by the question 'Does someone in your house own a car?' Household size was dichotomised into response options of 'four or less' and 'five or more'.

#### Diet

Participants were asked how many times a week they consumed soft drink, fruit juice, cordial, milk, tea, fruit and sweets, based on published literature (Levine, 2001; Jamieson et al., 2006; Jamieson et al., in press) [25-27]. Response options were dichotomised into 'every day or a few times per week' and 'once a week or less often'. Participants were additionally asked if they took sugar with their tea.

#### Dental service utilization

Participants were asked if they had visited a dentist before.

#### Dental behaviour

Participants were asked if they owned a toothbrush and, if so, if they brushed their teeth the previous day and at what age they had started to brush their teeth.

#### Dental fear

Participants were asked if they would feel scared about going to the dentist, with responses dichotomised into 'no' and 'little bit, fair bit or heaps'.

#### Clinical oral health indicators

Information about clinical oral health status was collected during standardised clinical examinations conducted by 2 calibrated dentists. Examining dentists followed a standardised protocol to record levels of tooth loss, dental decay experience and periodontal disease (for those with no medical contra-indications to periodontal probing). Oral mucosal lesions were also assessed. All dental diagnostic criteria were based on those employed in Australia's second national survey of adult oral health [22].

The DMFT (sum of decayed, missing and filled teeth in the permanent dentition) index was used to assess dental caries outcomes. All teeth present were divided into five tooth surfaces; occlusal/incisal, mesial, buccal, palatal/lingual and distal. Each dental surface was assessed and categorised using visual criteria only. Untreated dental decay was defined as 'cavitation of enamel or dentinal involvement or both being present' or 'visible caries that is contiguous with a restoration'. Filled due to decay was recorded when a tooth contained one or more permanent restorations placed to treat caries, while missing was recorded when a tooth had been extracted due to pathology. Experience of dental disease measures were considered as percent DMFT>0, percent DT>0, percent MT>0 and percent FT>0.

The US Centres for Disease Control and Prevention and the American Academy of Periodontology definitions were used to describe moderate and severe periodontal disease; whereby moderate periodontal disease was defined as the presence of either two sites between adjacent teeth with 4 mm+ attachment loss, or at least two such sites with 5 mm+ pockets. Severe periodontal disease was classified as having at least two sites between adjacent teeth with 6 mm+ attachment loss and with at least one 5 mm+ pocket [28].

Inflamed or abnormal mucosa was considered as one or more draining sinus, a suspected malignant tumour or ulcerated lesions (apthous, herpetic, traumatic).

Repeat examinations for examiner reliability were not possible due to logistical and time constraints imposed by the study's multidisciplinary nature.

### Data analytic approach

Univariate and bivariate distributions of severe oral health impact were determined. Correlation tests confirmed the existence of weak associations between items in a given group (Pearson's correlation coefficient range 0.1-0.4), with two variables needing to be excluded due to collinearity; consumption of tea (correlated with drinking sugar with tea; r = 0.44) and DMFT>0 (correlated with DT>; r = 0.89). Odds ratios of severe oral health impairment outcomes were determined using logistic regression modelling. Exposure variables were classified into demographic, socio-economic, diet, dental service utilisation, dental behaviour, dental fear and clinical oral health outcomes.

Three logistic regression models were constructed; Model A included non-clinical risk indicators, Model B included clinical risk indicators and Model C included both non-clinical and clinical risk indicators. The final regression model for the severe oral health impairment measure was constructed by removing covariates one at a time according to P-value size. Adjusted odds ratios were considered statistically significant when P-values derived from the Wald statistic were =0.05. Data were analysed using Intercooled STATA 8.

## Results

**Figure 1:**
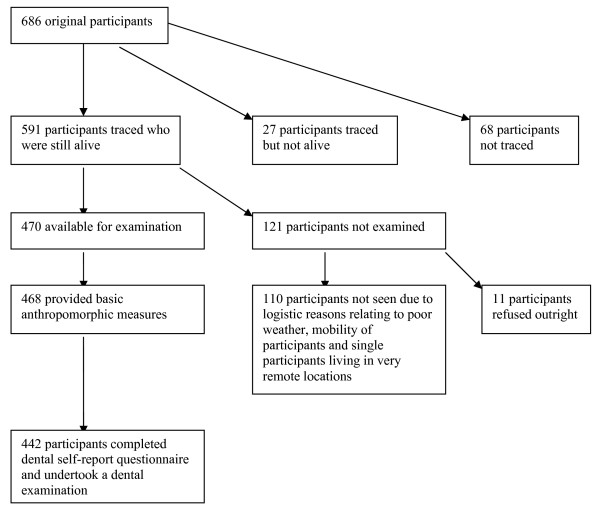
**Flow chart depicting ABC study Wave-3 participation in self-report dental questionnaire and dental examination**.

A flow chart depicting participation in the dental component of Wave-3 of the ABC study is presented (Figure 1). Four hundred and forty two participants aged 16-20 years completed a dental self-report interview and were clinically examined. Outcomes of the various components of the oral health impairment items are presented in Table 1. Just over one quarter of participants reported that they had trouble with their teeth, gum or jaw right now, 30 percent reported that, since the last wet, they had stopped eating some foods because of pain and almost two thirds reported that the believed that 'some or none' of their teeth 'looked ok'. The prevalence of the combination of any two oral health impairment items ranged from 17 percent (prevalence of toothache and avoidance eating food) to 66 percent (prevalence of avoidance eating food or dissatisfaction with appearance). Just over two thirds of participants reported one or more oral health impairment, while 16 percent had experienced all three oral health impairments (severe oral health impairment; Table 1).

**Table 1:** Prevalence of 16-20-year-old ABC study participants with experience of toothache/impaired appearance/food avoidance (95% CI in brackets)

Prevalence of toothache	26.2 (22.1-30.3)
Prevalence of avoidance eating food	30.5 (26.2-34.8)
Prevalence of dissatisfied appearance	63.8 (59.3-68.3)
Prevalence of toothache OR avoidance eating food	40.3 (35.8-44.8)
Prevalence of toothache AND avoidance eating food	16.5 (13.1-19.9)
Prevalence of toothache OR dissatisfied appearance	65.4 (61.0-69.8)
Prevalence of toothache AND dissatisfied appearance	24.7 (20.7-28.7)
Prevalence of avoidance eating food OR dissatisfied appearance	65.8 (61.4-70.2)
Prevalence of avoidance eating food AND dissatisfied appearance	28.5 (24.3-32.7)
Prevalence of impaired oral health summary measure_any	67.2 (62.8-71.6)
Prevalence of impaired oral health summary measure_all (severe oral health impairment)	16.3 (12.9-19.7)

A higher prevalence of toothache was noted among females; those aged 19-20 years; those reporting a household size of four or less people; soft drink, cordial or sweet consumption every day or a few days a week; non-ownership of a toothbrush; owning a toothbrush but not brushing the previous day; and experience of untreated dental decay (Table 2). Factors associated with food avoidance included soft drink or sweet consumption every day or a few days a week; fruit consumption once a week or less often; non-ownership of a toothbrush; reported commencement of toothbrushing upon eruption of permanent teeth; dental fear; experience of untreated dental caries; and inflamed or abnormal mucosa. There were nine factors associated with dissatisfaction with appearance; soft drink or sweet consumption every day or a few days per week; fruit consumption once a week or less often; non-ownership of a toothbrush; ownership of a toothbrush but not brushing the previous day; dental fear; experience of untreated dental decay; experience of restorations; and experience of moderate or severe periodontal disease. A higher prevalence of any oral health impairment component being reported was noted among those who consumed soft drink or sweets every day or a few days per week; non-ownership of a toothbrush; ownership of a toothbrush but not brushing the previous day; dental fear; experience of untreated dental caries; experience of restorations; and experience of moderate or severe periodontal disease. A higher prevalence of severe oral health impairment (reporting all three oral health impairment components) was noted among females; those aged 19-20 years; those who reported consuming soft drink or sweets every day or a few times a week; those who added sugar to their tea; non-ownership of a toothbrush; toothbrush ownership but not brushing the previous day; experience of untreated dental decay; and inflamed or abnormal oral mucosa.

**Table 2:** Total counts and prevalences of 16-20-year-old ABC study participants with experience of toothache/impaired appearance/food avoidance by demographic, socio-economic, diet, behavioural, dental service utilisation and clinical oral health variables (95% CI in brackets)

	**Counts**	**Prevalence of toothache**^a^	**Prevalence of avoid eating food**^b^	**Prevalence of dissatisfied appearance**^c^	**Prevalence of impaired oral health summary measure_any**^d^	**Prevalence of impaired oral health summary measure_all**^e^
						
**Total**	442	26.2 (22.1-30.3)	30.5 (26.2-34.8)	63.8 (59.3-68.3)	67.2 (62.8-71.6)	16.3 (12.9-19.7)
						
**Demographic**						
Sex						
Male	216	21.8 (16.3-27.3)*	26.9 (21.0-32.8)	60.6 (54.1-67.1)	66.2 (59.9-72.5)	12.0 (7.7-16.3)*
Female	226	30.5 (24.5-36.5)	34.1 (28.0-40.2)	66.8 (60.7-72.9)	68.1 (62.1-74.1)	20.4 (15.2-25.6)
						
Age-group						
16-18 years	301	21.9 (17.3-26.5)*	29.2 (24.1-34.3)	63.5 (58.1-68.9)	67.4 (62.1-72.7)	12.6 (8.9-16.3)*
19-20 years	141	35.5 (27.6-43.4)	33.3 (25.6-41.0)	64.5 (56.6-72.4)	66.7 (59.0-74.4)	24.1 (17.1-31.1)
						
Residential location						
Regional	94	30.9 (21.6-40.2)	24.5 (15.8-33.2)	64.9 (55.3-74.5)	68.1 (58.7-77.5)	17.0 (9.4-24.6)
Rural/remote	348	25.0 (20.5-29.5)	32.2 (27.3-37.1)	63.5 (58.5-68.5)	67.0 (62.1-71.9)	16.1 (12.3-19.9)
						
**Socio-economic**						
Source of household income						
Job	52	34.6 (21.7-47.5)	28.8 (16.6-41.0)	63.5 (50.5-76.5)	63.5 (50.5-76.5)	23.1 (11.7-34.5)
Welfare	390	25.1 (20.8-29.4)	30.8 (26.2-35.4)	63.8 (59.1-68.5)	67.7 (63.1-72.3)	15.4 (11.8-19.0)
						
Household size						
Four or less people	87	36.8 (26.7-46.9)*	32.2 (22.4-42.0)	69.0 (59.3-78.7)	73.6 (64.4-82.8)	19.5 (11.2-27.8)
Five or more people	355	23.7 (19.3-28.1)	30.1 (25.4-34.8)	62.5 (57.5-67.5)	65.6 (60.7-70.5)	15.5 (11.8-19.2)
						
Do you own a car?						
No	407	26.0 (21.8-30.2)	31.0 (26.5-35.5)	63.9 (59.3-68.5)	67.3 (62.8-71.8)	16.2 (12.6-19.8)
Yes	35	28.6 (13.7-43.5)	25.7 (11.3-40.1)	62.9 (47.0-78.8)	65.7 (50.1-81.3)	17.1 (4.7-29.5)
						
Someone in house own car?						
No	242	26.0 (20.5-31.5)	32.2 (26.3-38.1)	61.6 (55.5-67.7)	66.1 (60.2-72.0)	16.5 (11.8-21.2)
Yes	200	26.5 (20.4-32.6)	28.5 (22.3-34.7)	66.5 (60.0-73.0)	68.5 (62.1-74.9)	16.0 (10.9-21.1)
						
**Diet**						
Soft drink consumption						
Every day or a few times a week	305	31.5 (26.3-36.7)*	34.4 (29.1-39.7)*	70.5 (65.4-75.6)*	73.0 (68.0-78.0)*	20.7 (16.2-25.2)*
Once a week or less often	137	14.6 (8.7-20.5)	21.9 (15.0-28.8)	48.9 (40.6-57.2)	54.0 (45.7-62.3)	6.6 (2.5-10.7)
						
Fruit juice consumption						
Every day or a few times a week	320	27.5 (22.6-32.4)	30.9 (25.9-35.9)	65.0 (59.8-70.2)	67.5 (62.4-72.6)	17.8 (13.6-22.0)
Once a week or less often	122	23.0 (15.6-30.4)	29.5 (21.4-37.6)	60.7 (52.1-69.3)	66.4 (58.1-74.7)	12.3 (6.5-18.1)
						
Cordial consumption						
Every day or a few times a week	289	29.1 (23.9-34.3)*	31.8 (26.5-37.1)	65.1 (59.6-70.6)	68.9 (63.6-74.2)	18.0 (13.6-22.4)
Once a week or less often	153	20.9 (14.5-27.3)	28.1 (21.0-35.2)	61.4 (53.7-69.1)	64.1 (56.5-71.7)	13.1 (7.8-18.4)
						
Milk consumption						
Every day or a few times a week	306	26.1 (21.2-31.0)	27.5 (22.5-32.5)	61.8 (56.4-67.2)	65.0 (59.7-70.3)	15.7 (11.6-19.8)
Once a week or less often	136	26.5 (19.1-33.9)	37.5 (29.4-45.6)	68.4 (60.6-76.2)	72.1 (64.6-79.6)	17.6 (11.2-24.0)
						
Do take sugar with tea?						
Yes	366	27.0 (22.5-31.5)	32.0 (27.2-36.8)	64.2 (59.3-69.1)	67.5 (62.7-72.3)	17.8 (13.9-21.7)*
No	76	22.4 (13.1-31.7)	23.7 (14.2-33.2)	61.8 (50.9-72.7)	65.8 (55.2-76.4)	9.2 (2.7-15.7)
						
Fruit consumption						
Every day or a few times a week	288	24.0 (19.1-28.9)	26.4 (21.3-31.5)*	60.8 (55.2-66.4)*	64.6 (59.1-72.3)	14.9 (10.8-19.0)
Once a week or less often	154	30.5 (23.3-37.7)	38.3 (30.7-45.9)	69.5 (62.3-76.7)	72.1 (65.1-79.1)	18.8 (12.7-24.9)
						
Sweet consumption						
Every day or a few times a week	238	34.9 (28.9-40.9)*	36.6 (30.5-42.7)*	73.1 (67.5-78.7)*	75.6 (70.2-81.0)*	21.8 (16.6-27.0)*
Once a week or less often	204	16.2 (11.2-21.2)	23.5 (17.7-29.3)	52.9 (46.1-59.7)	57.4 (50.6-64.2)	9.8 (5.7-13.9)
						
**Dental service utilisation**						
Visited dentist before?						
Yes	412	26.9 (22.6-31.2)	30.1 (25.7-34.5)	63.6 (59.0-68.2)	66.7 (62.2-71.2)	16.7 (13.1-20.3)
No	30	16.7 (3.4-30.0)	36.7 (19.5-53.9)	66.7 (49.9-83.5)	73.3 (57.5-89.1)	10.0 (0-20.7)
						
**Dental behaviour**						
Toothbrush ownership						
Yes	303	22.4 (17.7-27.1)*	26.1 (21.2-31.0)*	58.4 (52.9-63.9)*	62.4 (57.0-67.8)*	12.9 (9.1-16.7)*
No	139	34.5 (26.6-42.4)	40.3 (32.2-48.4)	75.5 (68.4-82.6)	77.7 (70.8-84.6)	23.7 (16.7-30.7)
						
If yes, did brush teeth yesterday?						
Yes	220	20.0 (14.7-25.3)*	25.0 (19.3-30.7)	55.0 (48.5-61.5)*	59.1 (52.6-65.6)*	10.5 (6.5-14.5)*
No	85	30.6 (20.9-40.3)	31.8 (22.0-41.6)	68.2 (58.4-78.0)	71.8 (62.3-81.3)	21.2 (12.6-29.8)
If yes, what age when started to brush?						
When had little teeth	164	20.7 (14.5-26.9)	20.7 (14.5-26.9)*	54.9 (47.3-62.5)	59.1 (51.6-66.6)	10.4 (5.8-15.0)
When had big teeth	128	23.4 (16.1-30.7)	32.8 (24.7-40.9)	62.5 (54.2-70.8)	64.8 (56.6-73.0)	15.6 (9.3-21.9)
						
**Dental fear**						
Would you feel scared about going to the dentist?						
No	181	22.1 (16.1-28.1)	22.7 (16.6-28.8)*	53.6 (46.4-60.8)*	56.9 (49.7-64.1)*	13.3 (8.4-18.2)
Little bit, fair bit, heaps	261	29.1 (23.6-34.6)	36.0 (30.2-41.8)	70.9 (65.4-76.4)	74.3 (69.0-79.6)	18.4 (13.7-23.1)
						
**Clinical oral health outcomes**						
DT>0						
Yes	322	31.4 (26.4-36.4)*	38.5 (33.2-43.8)*	74.8 (70.1-79.5)*	78.3 (73.8-82.8)*	20.5 (16.1-24.9)*
No	120	12.5 (6.6-18.4)	9.2 (4.1-14.3)	34.2 (25.8-42.6)	37.5 (28.9-46.1)	5.0 (1.1-8.9)
						
MT>0						
Yes	241	28.6 (22.9-34.3)	29.0 (23.3-34.7)	65.1 (59.1-71.1)	67.2 (61.3-73.1)	17.8 (13.0-22.6)
No	201	23.4 (17.6-29.2)	32.3 (25.9-38.7)	62.2 (55.5-68.9)	67.2 (60.7-73.7)	14.4 (9.6-19.2)
						
FT>0						
Yes	99	29.3 (20.4-38.2)	34.3 (25.0-43.6)	72.7 (64.0-81.4)*	74.7 (66.2-83.2)*	16.2 (9.0-23.4)
No	343	25.4 (20.8-30.0)	29.4 (24.6-34.2)	61.2 (56.1-66.3)	65.0 (60.0-70.0)	16.3 (12.4-20.2)
						
Moderate or severe periodontal disease						
Yes	119	26.9 (19.0-34.8)	35.3 (26.8-43.8)	75.6 (67.9-83.3)*	77.3 (69.8-84.8)*	19.3 (12.2-26.4)
No	323	26.0 (21.2-30.8)	28.8 (23.9-33.7)	59.4 (54.1-64.7)	63.5 (58.3-68.7)	15.2 (11.3-19.1)
						
Inflamed/abnormal oral mucosa						
Yes	51	35.3 (22.3-48.3)	47.1 (33.5-60.7)*	70.6 (58.2-83.0)	76.5 (64.9-88.1)	27.5 (15.3-39.7)*
No	391	25.1 (20.8-29.4)	28.4 (24.0-32.8)	62.9 (58.1-67.7)	66.0 (61.3-70.7)	14.8 (11.3-18.3)

^a^A response of 'Yes' to the item 'Do you have any trouble with your teeth, gum or jaw right now?'. ^b^A response of 'Yes' to the item 'Since the last wet, have you stopped eating some foods because they hurt your teeth?'. ^a^A response of 'Some or none good' to the item 'Do you think your teeth are looking ok?'. ^d^A response of 'Yes' to the items 'Do you have any trouble with your teeth, gum or jaw right now?' OR 'Since the last wet, have you stopped eating some foods because they hurt your teeth?' OR a response of 'Some or none good' to the item 'Do you think your teeth are looking ok?'. ^e^A response of 'Yes' to the items 'Do you have any trouble with your teeth, gum or jaw right now?' AND 'Since the last wet, have you stopped eating some foods because they hurt your teeth?' AND a response of 'Some or none good' to the item 'Do you think your teeth are looking ok?'. *P < 0.05

In multivariate modelling, non-clinical risk indicators significantly associated with severe oral health impairment included being female, being aged 19-20 years; soft-drink consumption every day or a few days a week and non-ownership of a toothbrush (Table 3, Model A). Significant clinical risk indicators in Model B included untreated dental decay. In Model C, non-clinical and clinical risk factors that remained significantly associated with severe oral health impairment after adjusting for confounding included being female, being aged 19-20 years, soft drink consumption every day or a few days a week; non-ownership of a toothbrush and untreated dental decay.

**Table 3:** Logistic regression models of 16-20-year-old ABC study participants with experience of toothache/impaired appearance/food avoidance^a^

	***Model A - non-clinical*** **Odds Ratio (95% CI)**	***Model B - clinical*** **Odds Ratio (95% CI)**	***Model C - non-clinical and clinical*** **Odds Ratio (95% CI)**
**Demographic**			
Sex			
Female	2.1 (1.2-3.7)	-	2.0 (1.2-3.6)
Male (ref)	1.00	-	1.00
			
Age-group			
19-20 years	2.1 (1.2-3.6)	-	2.1 (1.2-3.6)
16-18 years (ref)	1.00	-	1.00
			
**Diet**			
Soft drink consumption			
Every day or a few times a week	3.2 (1.5-6.7)	-	2.6 (1.2-5.6)
Once a week or less often (ref)	1.00	-	1.00
			
**Dental behaviour**			
Toothbrush ownership			
No	2.0 (1.2-3.4)		1.9 (1.1-3.4)
Yes (ref)	1.00	-	1.00
			
**Clinical oral health outcomes**			
DT>0			
Yes	-	4.9 (2.1-11.6)	4.0 (1.6-9.6)
No (ref)	-	1.00	1.00
			

^a^A response of 'Yes' to the items 'Do you have any trouble with your teeth, gum or jaw right now?' AND 'Since the last wet, have you stopped eating some foods because they hurt your teeth?' AND a response of 'Some or none good' to the item 'Do you think your teeth are looking ok?'

## Discussion

This study set out to determine risk indicators for a summary measure of severe oral health impairment among a cohort of Australian Aboriginal young adults. Participants were described as 'young adults' because a number were aged 20 years, therefore could not be considered adolescent; and because many were parents, thus having responsibilities more aligned with young adults as opposed to adolescents [29]. It is notable that even when such a stringent definition of oral health impairment was employed, factors amenable to modification, such as soft drink consumption, toothbrush ownership and untreated dental decay, were associated with adverse impacts. The findings are supported by those of Levine et al. [30], who reported that factors most strongly associated with dental caries in their sample of English 11-15 year-olds included consumption of sugar-sweetened drinks and lack of regular tooth brushing.

We acknowledge that our definition of severe oral health impairment was stringent, with the criteria requiring the reporting of dental pain AND dissatisfaction with appearance AND difficulties eating. When establishing a case definition for severe impaired oral health, it is important that threshold values that constitute unequivocal evidence of impaired oral health at a given time are determined, in order to establish a true 'case' of severe oral health impairment that can then be monitored over time and particular after an intervention that aims to reduce this measure of oral health-related quality of life. There is a risk, when using less stringent measures - such as the presence of one or more of the oral health impairments - that severe oral health impairment is incorrectly categorised because these measures are less sensitive than the more strict case definition.

It was concerning that oral health impairments were reported so frequently in this cohort of young Indigenous Australians, particularly when it has been argued that Australians in this generation have had, on the whole, the best opportunities for good oral health among those born in the 20^th ^century [31]. Clearly Indigenous Australians in this birth cohort have not been privy to the same social and economic benefits that result in good oral health-related quality of life, an issue that rents at the very fabric of a society given evidence that oral health impairments impact on ability to enjoy social occasions, operate effectively in the home environment and function in the workplace/wider community [32].

The literature suggests that females frequently perceive the impacts of oral health impairments on quality of life as being greater than males [18,33-35]. Our finding reflects this generic trend, although the reasons behind it are less clear. Possible causes could relate to females in general having heightened perceptions of health impairments, including heightened perceptions of pain [36], impact on function [37] and quality of life [38].

It is perhaps intuitive that older age would be a risk indicator for severe oral health impairment, given population-level evidence in Australia that suggests that, up until middle age at least, increasing age is associated with poorer oral health-related quality of life [39]. Reasons include younger age-groups who experience oral dysfunction or discomfort being less likely to let it affect their psychological well-being, or a greater readiness among older populations to report severe impacts.

The literature is replete with examples of regular consumption of soft-drinks being associated with increased dental caries experience [40-42]. There is less evidence of the association between regular soft-drink consumption and impaired oral health. One possible causal pathway could involve the increased consumption of soft drink contributing to increased dental caries levels which, when untreated, lead to all three of the oral health impairment measures assessed in this study (that is, dental pain, dental dysfunction and issues with appearance).

Non-ownership of a toothbrush indicates little to no oral self-care on a regular basis. Poor oral hygiene is associated with tooth loss [43] and oral malodour [44], each of which has substantial social and psychological ramifications. There are a number of reasons why non-ownership of a toothbrush might be highly prevalent among the study population, including lack of availability from community stores, lack of affordability at community stores, limited storage areas in households, constant movement meaning toothbrushes are frequently misplaced, and general policies of sharing, meaning one does not necessarily 'own' a toothbrush.

It was intuitive that risk indicators for oral health impairment included clinical oral health outcomes such as decayed teeth, given the established relationship between dental disease outcomes, pain, difficulties eating, dissatisfaction with dental appearance and consequent impact on life quality [45]. The findings emphasize the importance of this group having regular access to culturally-sensitive oral health services that include the aesthetic treatment of dental decay. Indigenous Australians access dental care less frequently than their non-Indigenous counterparts, and usually do so because of pain [46].

It is important to examine limitations of the investigation. Although the study is longitudinal in design, important oral health impairment indicators were only collected in the most recent phase. True causality can therefore not be determined in this cross-sectional analysis, although this should be possible in future data collection waves. The self-report nature of the non-clinical items may have led to an under-estimation of these factors. However, we took great care with interviewing and, in any case, non-differential under-reporting would have resulted in more conservative estimates of the socio-demographic and oral health behaviour-related associations with oral health impairment, meaning our findings are unlikely to be spurious. Although the generalisability of the findings to the source population has not been established, Aboriginal people in Australia's National Survey of Adult Oral Health had markedly higher levels of oral health impact than their non-Aboriginal counterparts (Jamieson et al., in press) [47]. Among the study's strengths are the high follow-up rates in each wave of the investigation, meaning the prospective determination of severe oral health impairment (and the length of time over which the exposure data are collected), and the use of data on severe oral health impairment incidence as well as prevalence, should be possible in future data collection waves.

## Conclusions

Severe oral health impairment was prevalent among this population. The findings suggest that public health strategies that address prevention and treatment of dental disease, ownership of oral self-care devices and better dietary regulation are needed if severe oral health impairment among Indigenous Australian young adults is to be reduced.

## Competing interests

The authors declare that they have no competing interests.

## Authors' contributions

LMJ conducted the analysis and drafted the manuscript. KRT provided critical feedback throughout the study and through the writing of the manuscript. SMS was responsible for the original design of the ABC study and participated in the writing and completion of the manuscript. All authors read and approved the final manuscript.

## Pre-publication history

The pre-publication history for this paper can be accessed here:

http://www.biomedcentral.com/1472-6831/10/1/prepub

## References

[B1] Cohen LK Slade GD The emerging field of oral health-related quality of life outcomes research Measuring oral health and quality of life 1997 Chapel Hill: University of North Carolina, Dental Ecology 1 10

[B2] Locker D Slade GD Concepts of oral health, disease and quality of life Measuring oral health and quality of life 1997 Chapel Hill: University of North Carolina, Dental Ecology 11 24

[B3] Slade GD Inglehart M, Bagramian R Assessment of Oral Health Related Quality of Life Oral health related quality of life 2002 Carl Stream, IL: Quintessence Publishing Co. 29 46

[B4] Cohen LA Bonito AJ Akin DR Manski RJ Macek MD Edwards RR Cornelius LJ Toothache pain: a comparison of visits to physicians, emergency departments and dentists J Am Dent Assoc 2008 139 1205 1216 18762631 10.14219/jada.archive.2008.0336

[B5] Guzzi G Medicine forgets dentistry Lancet 2005 366 894 10.1016/S0140-6736(05)67316-3 16154015

[B6] Honkala E Honkala S Rimpelä A Rimpelä M The trend and risk factors of perceived toothache among Finnish adolescents from 1977 to 1997 J Dent Res 2001 80 1823 1827 10.1177/00220345010800091001 11926241

[B7] Ståhlnacke K Söderfeldt B Unell L Halling A Axtelius B Perceived oral health: changes over 5 years in one Swedish age-cohort Community Dent Oral Epidemiol 2003 31 292 299 10.1034/j.1600-0528.2003.00008.x 12846852

[B8] Meng X Gilbert GH Litaker MS Dynamics of satisfaction with dental appearance among dentate adults: 24-month incidence Community Dent Oral Epidemiol 2008 36 370 381 10.1111/j.1600-0528.2007.00409.x 19145724

[B9] Newton JT Prabhu N Robinson PG The impact of dental appearance on the appraisal of personal characteristics Int J Prosthodont 2003 16 429 434 12956500

[B10] Newton JT Minhas G Exposure to 'ideal' facial images reduces facial satisfaction: an experimental study Community Dent Oral Epidemiol 2005 33 410 418 10.1111/j.1600-0528.2005.00239.x 16262608

[B11] de Oliveira CM Sheiham A The relationship between normative orthodontic treatment need and oral health-related quality of life Community Dent Oral Epidemiol 2003 31 426 436 10.1046/j.1600-0528.2003.00002.x 14986910

[B12] Wong AH Cheung CS McGrath C Developing a short form of Oral Health Impact Profile (OHIP) for dental aesthetics: OHIP-aesthetic Community Dent Oral Epidemiol 2007 35 64 72 10.1111/j.1600-0528.2007.00330.x 17244139

[B13] Lee S McGrath C Samman N Quality of life in patients with dentofacial deformity: a comparison of measurement approaches Int J Oral Maxillofac Surg 2007 36 488 492 10.1016/j.ijom.2007.01.011 17339101

[B14] Llewellyn CD Warnakulasuriya S The impact of stomatological disease on oral health-related quality of life Eur J Oral Sci 2003 111 297 304 10.1034/j.1600-0722.2003.00057.x 12887394

[B15] Pace-Balzan A Butterworth CJ Dawson LJ Lowe D Rogers SN The further development and validation of the Liverpool Oral Rehabilitation Questionnaire (LORQ) version 3: a cross-sectional survey of patients referred to a dental hospital for removable prostheses replacement J Prosthet Dent 2008 99 233 242 10.1016/S0022-3913(08)60048-7 18319095

[B16] Pattussi MP Olinto MT Hardy R Sheiham A Clinical, social and psychosocial factors associated with self-rated oral health in Brazilian adolescents Community Dent Oral Epidemiol 2007 35 377 386 10.1111/j.1600-0528.2006.00339.x 17822486

[B17] Moynihan P The interrelationship between diet and oral health Proc Nutr Soc 2005 64 571 580 10.1079/PNS2005431 16313700

[B18] Harford J Spencer AJ Slade GD, Spencer AJ, Roberts-Thomson KF Oral health perceptions Australia's Dental Generations; The National Survey of Adult Oral Health 2004-2006. AIHW cat. no. DEN 165 2007 Canberra: Australian Institute of Health and Welfare 173 195

[B19] Budtz-Jorgensen E Chung JP Rapin CH Nutrition and oral health Best Pract Res Clin Gastroenterol 2001 15 885 896 10.1053/bega.2001.0247 11866483

[B20] Australian Bureau of Statistics Australian Census, 2006 Census Tables 2007 Canberra, ABS

[B21] Australian Bureau of Statistics The Health and Welfare of Australia's Aboriginal and Torres Strait Islander Peoples 2008 Canberra: Australian Bureau of Statistics

[B22] Slade GD Spencer AJ Roberts-Thomson KF editors Australia's Dental Generations; The National Survey of Adult Oral Health 2004-2006. AIHW cat. no. DEN 165 2007 Canberra: Australian Institute of Health and Welfare

[B23] Brennan DS Roberts-Thomson KF Spencer AJ Oral health of Indigenous adult public dental patients in Australia Aust Dent J 2007 52 322 328 10.1111/j.1834-7819.2007.tb00509.x 18265689

[B24] Sayers SM Mackerras D Singh G Bucens I Flynn K Reid A An Australian Aboriginal birth cohort: a unique resource for a life course study of an Indigenous population. A study protocol BMC Int Health Hum Rights 2003 3 1 10.1186/1472-698X-3-1 12659639 PMC152651

[B25] Levine RS Caries experience and bedtime consumption of sugar-sweetened food and drinks--a survey of 600 children Community Dent Health 2001 18 228 31 11789700

[B26] Jamieson LM Bailie RS Beneforti M Koster CR Spencer AJ Dental self-care and dietary characteristics of remote-living Indigenous children Rur Rem Health 2006 6 503 16646637

[B27] Jamieson LM Roberts-Thomson KF Sayers SM Dental caries risk indicators among Australian Aboriginal young adults Community Dent Oral Epidemiol in press 10.1111/j.1600-0528.2009.00519.x 20059488

[B28] Page RC Eke PI Case definitions for use in population-based surveillance of periodontal disease J Periodontol 2007 78 1387 1399 10.1902/jop.2007.060264 17608611

[B29] Sayers SM Singh G Mackerras D Lawrance M Gunthorpe W Jamieson L Davison B Schutz K Fitz J Australian Aboriginal Birth Cohort study: follow-up processes at 20 years BMC Int Health Human Rights 2009 9 23 10.1186/1472-698X-9-23 PMC2761846 19775475

[B30] Levine RS Nugent ZJ Rudolf MC Sahota P Dietary patterns, toothbrushing habits and caries experience of schoolchildren in West Yorkshire, England Community Dent Health 2007 24 82 87 17615822

[B31] Do LG Roberts-Thomson KF Dental caries experience in the Australian adult population Aust Dent J 2007 52 249 251 10.1111/j.1834-7819.2007.tb00496.x 17969295

[B32] John MT Hujoel P Miglioretti DL LeResche L Koepsell TD Micheelis W Dimensions of oral-health-related quality of life J Dent Res 2004 83 956 960 10.1177/154405910408301213 15557405

[B33] Acharya S Oral health-related quality of life and its associated factors in an Indian adult population Oral Health Prev Dent 2008 6 175 184 19119571

[B34] Kelly M Steele J Nuttall N Bradnock G Morris J Nunn J Adult dental health survey: oral health in the United Kingdom 1998 2000 London: The Stationery Office

[B35] Mason J Pearce MS Walls AW Parker L Steele JG How do factors at different stages of the lifecourse contribute to oral-health-related quality of life in middle age for men and women? J Dent Res 2006 85 257 61 10.1177/154405910608500310 16498074

[B36] Bood SA Kjellgren A Norlander T Treating stress-related pain with the flotation restricted environmental stimulation technique: are there differences between women and men? Pain Res Manag 2009 14 293 298 19714269 10.1155/2009/298935 PMC2734516

[B37] Gammage KL Gabriel DA Trait self-presentational concerns and performance in a maximal isometric strength test J Strength Cond Res 2009 23 1287 1291 19528846 10.1519/JSC.0b013e31819f1e40

[B38] Norris CM Spertus JA Jensen L Johnson J Hegadoren KM Ghali WA Sex and gender discrepancies in health-related quality of life outcomes among patients with established coronary artery disease Circ Cardiovasc Qual Outcomes 2008 1 123 130 10.1161/CIRCOUTCOMES.108.793448 20031799

[B39] Slade GD Nuttall N Sanders AE Steele JG Allen PF Lahti S Impacts of oral disorders in the United Kingdom and Australia Br Dent J 2005 198 489 493 10.1038/sj.bdj.4812252 15849587

[B40] Llena C Forner L Dietary habits in a child population in relation to caries experience Caries Res 2008 42 387 393 10.1159/000154784 18781067

[B41] Burt BA Kolker JL Sandretto AM Yuan Y Sohn W Ismail AI Dietary patterns related to caries in a low-income adult population Caries Res 2006 40 473 480 10.1159/000095645 17063017 PMC1626651

[B42] Shenkin JD Heller KE Warren JJ Marshall TA Soft drink consumption and caries risk in children and adolescents Gen Dent 2003 51 30 36 15061331

[B43] Kressin NR Boehmer U Nunn ME Spiro A Increased preventive practices lead to greater tooth retention J Dent Res 2003 82 223 227 10.1177/154405910308200314 12598553

[B44] Quirynen M Zhao H van Steenberghe D Review of the treatment strategies for oral malodour Clin Oral Investig 2002 6 1 10 11996157 10.1007/s00784-002-0152-9

[B45] Selwitz RH Ismail AI Pitts NB Dental caries Lancet 2007 369 51 59 10.1016/S0140-6736(07)60031-2 17208642

[B46] Spencer AJ Harford J Slade GD, Spencer AJ, Roberts-Thomson KF Dental Care Australia's Dental Generations; The National Survey of Adult Oral Health 2004-2006. AIHW cat. no. DEN 165 2007 Canberra: Australian Institute of Health and Welfare 143 172

[B47] Jamieson LM Mejia GC Slade GD Roberts-Thomson KF Risk factors for impaired oral health among 18-34-year-old Australians J Pub Health Dent in press 10.1111/j.1752-7325.2009.00151.x 19780909

